# Approved Societies and Hospital Treatment

**Published:** 1922-06

**Authors:** F. Squires


### Approved Societies and Hospital Treatment.

*To the Editor of* The Hospital and Health Review.

Sir, — With reference to my article, may I say that the scheme referred to is the one arranged by the British Hospitals Association with the Approved Societies, and I am indebted to the Association for the main facts contained in the article.

Yours faithfully,

F. Squires.

